# Course of Early Neurologic Symptom Severity after Endovascular Treatment of Anterior Circulation Large Vessel Occlusion Stroke: Association with Baseline Multiparametric CT Imaging and Clinical Parameters

**DOI:** 10.3390/diagnostics11071272

**Published:** 2021-07-15

**Authors:** Matthias Philipp Fabritius, Teresa A. Wölfer, Moriz Herzberg, Steffen Tiedt, Daniel Puhr-Westerheide, Sergio Grosu, Stefan Maurus, Thomas Geyer, Adrian Curta, Lars Kellert, Clemens Küpper, Thomas Liebig, Jens Ricke, Konstantinos Dimitriadis, Wolfgang G. Kunz, Hanna Zimmermann, Paul Reidler

**Affiliations:** 1 Department of Radiology, University Hospital, LMU Munich, Marchioninistr. 15, 81377 Munich, Germany; daniel.puhr-westerheide@med.uni-muenchen.de (D.P.-W.); Sergio.Grosu@med.uni-muenchen.de (S.G.); stefan.maurus@med.uni-muenchen.de (S.M.); thomas.geyer@med.uni-muenchen.de (T.G.); adrian.curta@med.uni-muenchen.de (A.C.); Jens.Ricke@med.uni-muenchen.de (J.R.); wolfgang.kunz@med.uni-muenchen.de (W.G.K.); paul.reidler@med.uni-muenchen.de (P.R.); 2 Institute for Stroke and Dementia Research, University Hospital, LMU Munich, Marchioninistr. 15, 81377 Munich, Germany; Teresa.Woelfer@med.uni-muenchen.de (T.A.W.); Steffen.Tiedt@med.uni-muenchen.de (S.T.); Konstantin.Dimitriadis@med.uni-muenchen.de (K.D.); 3 Department of Diagnostic and Interventional Radiology, University Hospital Würzburg, Josef-Schneider-Straße 2, 97080 Würzburg, Germany; Herzberg_M1@ukw.de; 4 Institute of Neuroradiology, University Hospital, LMU Munich, Marchioninistr. 15, 81377 Munich, Germany; Thomas.Liebig@med.uni-muenchen.de (T.L.); Hanna.Zimmermann@med.uni-muenchen.de (H.Z.); 5 Department of Neurology, University Hospital, LMU Munich, Marchioninistr. 15, 81377 Munich, Germany; Lars.Kellert@med.uni-muenchen.de (L.K.); Clemens.Kuepper@med.uni-muenchen.de (C.K.)

**Keywords:** stroke, large vessel occlusion, multiparametric CT, CT perfusion, CT angiography, NIHSS, EVT

## Abstract

Background: Neurologic symptom severity and deterioration at 24 hours (h) predict long-term outcomes in patients with acute large vessel occlusion (LVO) stroke of the anterior circulation. We aimed to examine the association of baseline multiparametric CT imaging and clinical factors with the course of neurologic symptom severity in the first 24 h after endovascular treatment (EVT). Methods: Patients with LVO stroke of the anterior circulation were selected from a prospectively acquired consecutive cohort of patients who underwent multiparametric CT, including non-contrast CT, CT angiography and CT perfusion before EVT. The symptom severity was assessed on admission and after 24 h using the 42-point National Institutes of Health Stroke Scale (NIHSS). Clinical and imaging data were compared between patients with and without early neurological deterioration (END). END was defined as an increase in ≥4 points, and a significant clinical improvement as a decrease in ≥4 points, compared to NIHSS on admission. Multivariate regression analyses were used to determine independent associations of imaging and clinical parameters with NIHSS score increase or decrease in the first 24 h. Results: A total of 211 patients were included, of whom 38 (18.0%) had an END. END was significantly associated with occlusion of the internal carotid artery (odds ratio (OR), 4.25; 95% CI, 1.90–9.47) and the carotid T (OR, 6.34; 95% CI, 2.56–15.71), clot burden score (OR, 0.79; 95% CI, 0.68–0.92) and total ischemic volume (OR, 1.01; 95% CI, 1.00–1.01). In a comprehensive multivariate analysis model including periprocedural parameters and complications after EVT, carotid T occlusion remained independently associated with END, next to reperfusion status and intracranial hemorrhage. Favorable reperfusion status and small ischemic core volume were associated with clinical improvement after 24 h. Conclusions: The use of imaging parameters as a surrogate for early NIHSS progression in an acute LVO stroke after EVT reached limited performance with only carotid T occlusion as an independent predictor of END. Reperfusion status and early complications in terms of intracranial hemorrhage are critical factors that influence patient outcome in the acute stroke phase after EVT.

## 1. Introduction

The National Institutes of Health Stroke Scale (NIHSS) represents the reference standard for the functional assessment of symptom severity in the setting of acute ischemic stroke [1,2,3]. Together with multiparametric CT or MRI imaging, it guides therapy decision making toward intravenous thrombolysis (IVT) and endovascular treatment (EVT) [4]. Early neurological deterioration (END) is commonly defined as increase in ≥4 points of the NIHSS between pretreatment (NIHSS on admission) and day one (24-h NIHSS) [5,6,7]. END occurs in 10–40% of patients, depending on different definitional sub-criteria and/or inclusion criteria [5,6,7,8,9,10]. However, studies have independently shown that the affected patients have a significantly worse functional outcome. Thus, an early NIHSS course serves as a useful prognostic parameter in stroke management, which was also demonstrated by a post hoc analysis of the REVASCAT trial [11]. The reasons for END can be rather obvious, such as infarct expansion, edema, hemorrhagic transformation, therapy-associated complications, or lack of reperfusion—where there is clearly causal crossover between one another — but unclear causes, so-called unexplained END (unEND), are also described [12,13]. A recent study found that mainly non-modifiable factors, such as a premorbid modified Rankin Scale score (mRS), diabetes mellitus and age, predict unEND in patients after EVT; however, baseline imaging parameters were not included in the analysis [5]. Overall, the 24-h NIHSS has been proven as a good predictor for long-term stroke outcome in a large multicentric study and is, therefore, a promising parameter to determine therapeutic efficacy early. However, knowledge of the influencing factors is crucial to pave the way for its use as an endpoint in future studies. As the most commonly performed imaging modality in acute stroke setting, multiparametric CT provides the opportunity to comprehensively assess the cerebrovascular status, including tissue perfusion, topography, collateral flow, or initial edema formation. These parameters can be directly translated to morphological correlates of stroke, for example penumbra and core volume, as well as the temporal course of infarct growth [14,15,16,17,18]. Recently, it has been shown that multiparametric CT-based total ischemic volume and clot burden influence the baseline NIHSS in acute large vessel occlusion (LVO) stroke and, thus, might serve as surrogate parameters for stroke symptom severity [19].

In this study, we aimed to determine the association of baseline multiparametric CT imaging and clinical factors with the course of neurological symptoms in the first 24 h after endovascular treatment (EVT).

## 2. Materials and Methods

### 2.1. Study Design and Cohort

This retrospective study was approved by the local institutional review board (LMU Munich) according to the Declaration of Helsinki of 2013 and the requirement for written informed consent was waived. Patients with an acute ischemic stroke due to anterior circulation LVO were selected out of a consecutive cohort of prospectively enrolled patients who were treated with EVT at our institution between 2015 and 2020 (German Stroke Registry; clinicaltrials.gov identifier: NCT03356392, approval date: 22 November 2017).

For our retrospective analysis, we included patients with the following:

- Stroke due to anterior circulation large vessel occlusion (internal carotid artery (ICA), middle cerebral artery (MCA));
- A complete imaging dataset on admission, including non-contrast CT (NCCT), CT angiography (CTA) and CT perfusion (CTP);
- Follow-up imaging (NCCT or MRI) within the first 24 h after EVT;
- A recorded 24-h NIHSS.

We excluded patients with the following:

- premorbid mRS ≥ 2.

### 2.2. Multiparametric CT Imaging Analysis

All patients underwent a standardized multiparametric CT protocol including NCCT, CTA and CTP. Examinations were performed on CT scanners of the same vendor (SOMATOM Force, Definition AS+ and Definition Flash, Siemens Healthineers, Forchheim, Germany). CTP data were processed using Syngo Neuro Perfusion CT (Siemens Healthineers, Forchheim, Germany), including automated calculation of ischemic core and penumbra volumes according to the manufacturer’s thresholds (CBV < 1.2 mL/100 mL and CBF < 35.1 mL/100 mL/min) [20]. The summated volumes of ischemic core and penumbra represent the total ischemic volume. CTA imaging was obtained in a single sweep from the aortic arch to the vertex with a bolus trigger of 100 HU in the aortic trunk. Expert readers (M.P.F. and P.R.), blinded to all clinical data, determined early ischemic changes using the Alberta Stroke Program Early CT Score (ASPECTS) on NCCT and CTA [21,22]. The occlusion location, regional leptomeningeal collateral (rLMC) score according to Menon et al. (20-point ordinal score, high values indicate good collaterals) and clot burden score according to Tan et al. (10-point ordinal score, small values indicate severe vessel occlusion) were determined on CTA [15,18]. Follow-up images were evaluated regarding the occurrence of peri- and post-procedural complications in the sense of parenchymal hematoma, other intracranial hemorrhages (other ICH) and space-occupying edemas [23,24]. Other ICHs included subarachnoid, subdural and other intracerebral bleeding not directly related to the infarcted tissue.

### 2.3. Functional and Clinical Parameters

Baseline demographic and clinical characteristics were recorded. All eligible patients without contraindications received intravenous thrombolysis followed by EVT. Reperfusion success was assessed using the modified treatment in cerebral infarction (mTICI) scale and defined as TICI 2b-3 [25,26]. NIHSS was determined for all patients on admission and, after twenty-four hours, via in-person examination by on call neurologists. END was defined as an increase in the NIHSS score of at least 4 points or death between baseline (NIHSS on admission) and day 1 (24-h NIHSS) of the ischemic event. Improvement in symptom severity after 24 h was defined as a decrease in the NIHSS score ≥4 compared to baseline. Baseline comorbidities including premorbid mRS and cardiovascular risk factors were systematically determined after initial therapy by patient interview or from medical records. The 90-day mRS score, as a functional outcome parameter, was assessed 90 days after the stroke event [27].

### 2.4. Endovascular Treatment

All patients were treated on a biplane angiography system (Artis Q, Siemens Healthineers, Forchheim, Germany) with continuous anesthesia support, applying either conscious sedation (CS) or general anesthesia (GA) and maintaining sufficient mean arterial blood pressure. The decision between CS and GA was made in consensus between the interventional neuroradiologist, the neurologist on call and the anesthesiologist according to foreseeable patient compliance and patient cardiopulmonary status with conscious sedation as the preferred approach [28]. A transfemoral access with the co-axial use of 8F guiding catheters and intermediate catheters (usually SOFIA, MicroVention, Tustin, CA, USA or Catalyst, Stryker, Fremont, CA, USA) was the standard of care, aiming at direct thrombus aspiration first, which—if unsuccessful—was converted to stent-retriever based thrombectomy with various approved stent retrievers (Solitaire, Trevo, Preset).

### 2.5. Statistical Analysis

Statistical analysis was performed using SPSS (SPSS Statistics 23, IBM, Armonk/NY 2016, USA). Categorical variables are presented as the count (percentages). Ordinal and continuous variables are presented as the median (interquartile range, IQR). To search for predictors of END, we compared patients without END to those with END. Univariate data analysis was performed using the Χ^2^ test for categorical and the Mann–Whitney U test for ordinal or continuous variables. Multivariate binary logistic regressions were performed, including baseline imaging parameters with *p* < 0.05 in univariate analysis, adjusted for age, sex and NIHSS on admission. Further, comprehensive multivariate analysis models, which additionally consider the influence of reperfusion status and peri- and postprocedural complications on the 24-h NIHSS, were performed to determine the independent associations of imaging and clinical parameters with an NIHSS score increase or decrease in the first 24 h. To avoid the overfitting of the regression models, the multicollinearity of independent variables was tested using the variance inflation factor. The *p* values below 0.05 were considered to indicate statistical significance.

## 3. Results

### 3.1. Patient Characteristics

**Figure 1:**
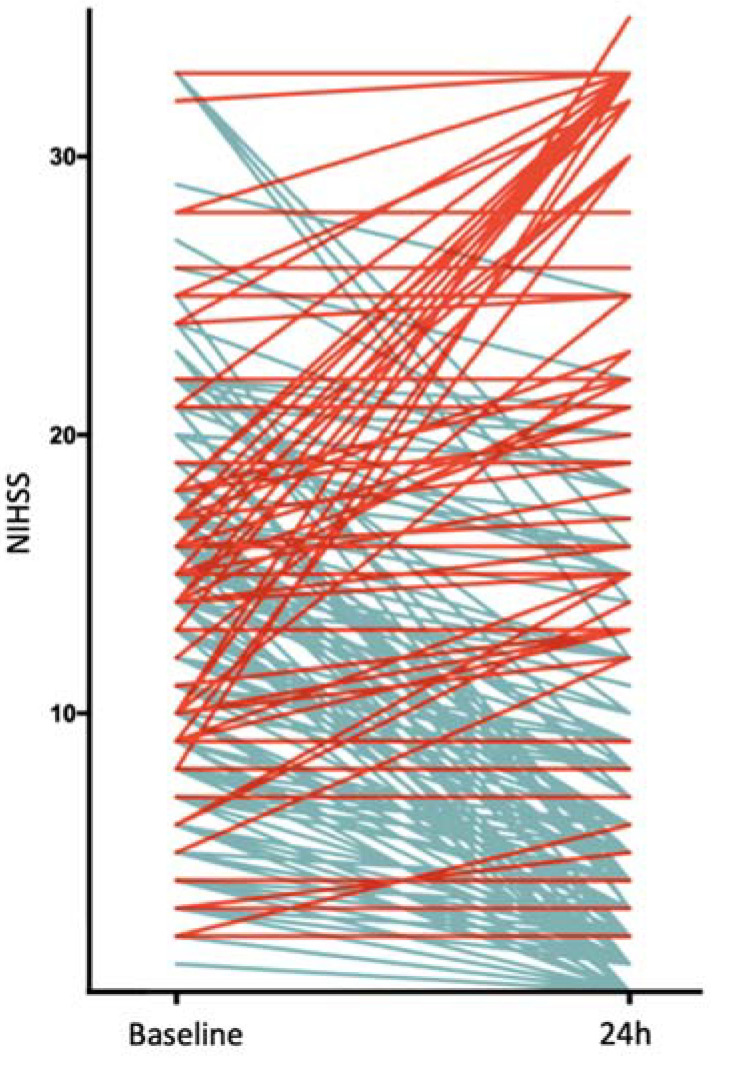
Early Course of NIHSS. Each line represents a patient. Red lines indicate an increase in NIHSS, blue lines indicate a decrease in NIHSS Abbreviations: NIHSS, National Institutes of Health Stroke Scale.

Among the 211 patients who matched the inclusion criteria for this retrospective analysis, 38 (18.0%) had an END. Of those, 24 (63.2%) patients died within 90 days after their stroke. All the patients were treated with EVT, 131 patients (62.1%) received additional intravenous thrombolysis. The median NIHSS score on admission was 14 in both groups, whereas the median 24-h NIHSS score differed significantly, with 32 (IQR:22–33) in the END group versus 6 (2–15) in the group without END. Figure 1 illustrates the course of the NIHSS score between the baseline and 24 h of all the patients. The patients with END exhibited lower clot burden scores (*p* = 0.049), a higher incidence of ICA (*p* = 0.002) and carotid T (*p* < 0.001) involvement and greater total ischemic volumes (*p* = 0.02). Furthermore, they had less favorable reperfusion status (<0.001) and more complications. There were no statistically significant differences between the two groups regarding early ischemic changes, time from symptom onset or cardiovascular risk factors. Patient and treatment characteristics, according to the occurrence of END or not, are reported in Table 1. The other ICHs included 14 subarachnoid hemorrhages (7 in each group), 2 subdural hematomas (both in the group without END), 3 intracerebral hemorrhages (2 in the group without END) and 1 intraventricular hemorrhage (END group). A total of 13 (6.2%) patients had an unEND (favorable recanalization and absence of peri- or postprocedural complications).

### 3.2. Association of END with Baseline Imaging and Clinical Parameters

In the multivariable binary logistic regression analysis of the baseline imaging parameters adjusted for age, sex and NIHSS on admission, a significant association with END was indicated for the ICA (OR, 4.25; 95% CI, 1.90–9.47) and carotid T (OR, 6.34; 95% CI, 2.56–15.71) involvement, the clot burden score (OR, 0.79; 95% CI, 0.68–0.92) and total ischemic volume (OR, 1.01; 95% CI, 1.00–1.01) (Table 2). In the comprehensive model, all the baseline imaging parameters, except for carotid-T occlusion (OR, 4.72; 95% CI, 1.39–16.10), were outperformed by the favorable reperfusion status (OR, 0.23; 95% CI, 0.09–0.59) and other ICHs (OR, 4.83; 95% CI, 1.45–16.02). The detailed results are displayed in Table 3.

### 3.3. Association of 24-h NIHSS Improvement with Baseline Imaging and Clinical Parameters

Favorable reperfusion status (OR, 3.22; 95% CI, 1.25–8.26) and small ischemic core volume (OR, 0.99; 95% CI, 0.97–1.00) were associated with an improvement in symptom severity after 24 h. The detailed results are displayed in Table 4.

## 4. Discussion

This study presents a comprehensive investigation of multiparametric CT imaging and clinical factors on neurological symptom severity progression in the first 24 h in anterior circulation LVO stroke. END occurred in 18.0% of the analyzed patients and was associated with a poor functional outcome. To date, only a few studies have investigated END after EVT with varying incidence rates and predictors of END due to different definition and inclusion criteria [5,8,10]. Girot et al., who focused on unEND, mainly found nonmodifiable factors such as premorbid disability (mRS ≥ 2), age and diabetes mellitus as predictors for neurologic deterioration; however, baseline imaging parameters were not included in the analysis and only cases with favorable reperfusion status and no early complications were considered as END, which limits the comparability with our results [5]. Kim et al. and Zhang et al. identified the recanalization status as the most important predictor [8,10]. Our results indicate that, besides the expected influencing factors of poor reperfusion and peri- and early postprocedural complications, the occlusion location is a determinant for END. This coincides with recently published data of a study that only examined minor stroke patients (NIHSS on admission < 6) treated with IVT [6]. Occlusions of the carotid T, which were identified as an independent risk factor for END in our cohort, cause among the most severe stroke syndromes, as they are associated with large ischemic areas and poor chronic functional outcomes [29]. It was previously shown that the total ischemic volume and collateral status correlate with the admission NIHSS and a linear relationship between the 24-h NIHSS and the post-EVT infarction volume was demonstrated, hinting toward similar mechanisms moderating symptom severity in the hyperacute and post-therapeutic acute stroke phase [11,30,31]. However, we could not find a correlation between initial ischemic volumes and END, which confirms EVT as an effective treatment method even in large infarcts with poor collateral status. Nevertheless, we were able to show that smaller infarct core volumes had a positive influence on NIHSS progression, indicating that a favorable initial perfusion status and sufficient reperfusion are crucial for a swift clinical improvement. Neither age nor baseline comorbidities exhibited a significant influence on the early NIHSS progression in our cohort, although association with baseline NIHSS has been established [19,30]. This is consistent with the knowledge that both older and younger patients benefit from EVT [32,33,34]. It is important to note that only 6.2% of the patients in our cohort had an unEND comparable to previous studies. This underlines the fact that only a few patients deteriorate in the first 24 h after favorable recanalization in the absence of peri- or postprocedural complications [5]. Accordingly, reperfusion status after EVT was the decisive factor for a significant worsening or improvement of symptoms within the first 24 h in our analyses.

There are limitations to this study that need to be considered when interpreting the results. First, it is a retrospective analysis of a selected single center database. We excluded patients with premorbid disability (premorbid mRS ≥ 2) as we wanted to examine the temporal effects on stroke symptom progression without the bias of existing disabilities. Due to the relatively small group size and the retrospective design, a sufficient subgroup analysis providing reliable findings regarding the influence of imaging parameters on unEND was not possible, nevertheless we believe that a comprehensive approach is also informative. Furthermore, to ensure data completeness, only the cases of END occurring within the first 24 h after stroke onset were taken into account, even though it is known that END can also occur a few days after the initial event [35,36]. The cut-off for neurological improvement was arbitrary, following the definition of END. To our knowledge, there are no established criteria for early neurological improvement by means of the NIHSS. Moreover, imaging was performed with scanners and software from a single vendor. Although the volumetric measures may differ between different software packages, the measurements of the package used in our series (Siemens Healthineers, Forchheim, Germany) previously showed the best agreement with the reference standard RAPID among other packages [20,37].

## Figures and Tables

**Table 1:** Patient characteristics.

	Overall (*n* = 211)		Without END (*n* = 173)		With END (*n* = 38)		*p* Value
Patient data							
Age	75	(63–81)	74	(63–81)	78	(72–80)	0.20
Female	92	(43.6%)	75	(43.4%)	17	(44.7%)	0.88
Male	119	(56.4%)	98	(56.6%)	21	(55.3%)	
Time from symptom onset to imaging (min) *	122	(69–214)	122	(69–210)	125	(65–238)	0.95
NIHSS on admission	14	(8–18)	14	(8–19)	14	(10–17)	0.85
**NCCT imaging data**							
ASPECTS NCCT	8	(7–10)	8	(7–10)	9	(6–10)	0.63
**CTA**							
Location of occlusion							
ICA	62	(29.4%)	43	(24.9%)	19	(50.0%)	**0.002**
Carotid T	45	(21.3%)	28	(16.2%)	17	(44.7%)	**<0.001**
M1 segment of MCA	165	(78.2%)	138	(79.8%)	27	(71.1%)	0.24
M2 segment of MCA	59	(28.5%)	50	(29.6%)	9	(23.7%)	0.47
Clot burden score	6	(4–8)	6	(4–8)	4	(2–8)	**0.049**
rLMC score	15	(12–18)	15	(12–18)	13	(10–17)	0.06
ASPECTS CTA	7	(5–8)	7	(5–8)	8	(5–8)	0.80
**CTP imaging data**							
Total ischemic volume (mL)	190	(154–239)	187	(152–230)	205	(179–271)	**0.02**
Ischemic core volume (mL)	35	(21–57)	33	(21–54)	50	(21–69)	0.06
Mismatch ratio	5.3	(3.6–7.8)	5.4	(3.7–7.8)	4.6	(3.5–7.0)	0.33
**Complications**							
Space-occupying edema	24	(11.4%)	16	(9.2%)	8	(21.1%)	**0.04**
Parenchymal hematoma	13	(6.2%)	8	(4.6%)	5	(13.5%)	**0.048**
Other ICH	18	(8.5%)	10	(5.8%)	8	(21.1%)	**0.002**
**Treatment data**							
Intravenous thrombolysis	131	(62.1%)	107	(61.8%)	24	(63.2%)	0.88
Favorable mTICI (≥2 b)	40	(18.9%)	22	(12.7%)	18	(47.4%)	**<0.001**
**Functional outcome data**							
24-h NIHSS	8	(3–19)	6	(2–15)	32	(22–33)	**<0.001**
90-day mRS *	3	(1–6)	2	(1–4)	6	(5–6)	**<0.001**
**Cardiovascular risk factors**							
Arterial hypertension	148	(70.1%)	117	(67.6%)	31	(81.6%)	0.09
Diabetes mellitus	25	(12.0%)	20	(11.6%)	5	(13.5%)	0.75
Hypercholesterolemia	45	(21.4%)	37	(21.4%)	8	(21.6%)	0.98
Atrial fibrillation	59	(28.1%)	52	(30.2%)	7	(18.4%)	0.14
Smoking history	35	(16.6%)	27	(15.6%)	8	(21.1%)	0.17

Values presented are no (percentage) for categorical and median (interquartile range) for ordinal and continuous variables. Proportion analysis tests for categorical variables were performed using *t*-test. Nonparametric tests for ordinal and continuous variables were performed using the Mann–Whitney U test. Bold *p* values indicate *p* < 0.05. Abbreviations: ASPECTS, Acute Stroke Prognosis Early CT Score; CTA, CT angiography; CTP, CT perfusion; END, Early Neurological Deterioration; ICA, Internal Carotid Artery; ICH, Intracranial hemorrhage; MCA, Middle Cerebral Artery; mRS, modified Rankin Scale; mTICI, modified Thrombolysis in Cerebral Infarction; NCCT, non-contrast CT; NIHSS, National Institutes of Health Stroke Scale; rLMC, regional leptomeningeal collateral. END was defined as 24-h increase in NIHSS score ≥4. * Missing values: Time from symptom onset 85/211 (18 in group with END); 90-day mRS 11/200 (0 in group with END).

**Table 2:** Multivariate analysis of baseline imaging predictors for END.

	END	
Independent Variables	OR (95% CI)	*p* Value
ICA occlusion	4.25 (1.90–9.47)	**<0.001**
Carotid T occlusion	6.34 (2.56–15.71)	**<0.001**
Clot burden score	0.79 (0.68–0.92)	**0.002**
Total ischemic volume	1.01 (1.00–1.01)	**0.005**

Multivariate binary logistic regression analyses were performed for the indicated parameters adjusted for age, sex and NIHSS on admission. END was defined as 24-h increase in NIHSS score ≥4 compared to baseline; Bold *p* values indicate *p* < 0.05. Abbreviations: see Table 1; CI, confidence interval; OR, odds ratio.

**Table 3:** Comprehensive multivariate analysis model for END.

	END	
Independent Variables	OR (95% CI)	*p* Value
Age	1.02 (0.98–1.06)	0.28
Sex	0.8 (0.31–1.81)	0.53
Carotid T occlusion	4.72 (1.39–16.10)	**0.013**
Clot burden score	1.09 (0.87–1.35)	0.46
rLMC score	0.94 (0.84–1.06)	0.32
Ischemic core volume	1.00 (0.99–1.01)	0.76
Mismatch ratio	1.02 (0.90–1.17)	0.73
Favorable mTICI (≥2 b)	0.23 (0.09–0.59)	**0.002**
Space-occupying edema	1.10 (0.32–3.71)	0.89
Parenchymal hematoma	2.18 (0.85–5.60)	0.11
Other ICH	4.83 (1.45–16.02)	**0.010**

Multivariate binary logistic regression analyses were performed for the indicated parameters. END was defined as 24-h increase in NIHSS score ≥4 compared to baseline; Bold *p* values indicate *p* < 0.05. Abbreviations: see Table 1; CI, confidence interval; OR, odds ratio.

**Table 4:** Comprehensive multivariate analysis model for 24-h NIHSS improvement.

	Improvement	
Independent Variables	OR (95% CI)	*p* Value
Age	0.98 (0.96–1.00)	0.08
Sex	0.66 (0.34–1.23)	0.20
Carotid T occlusion	1.02 (0.36–2.88)	0.97
Clot burden score	0.95 (0.80–1.12)	0.53
rLMC score	0.97 (0.89–1.06)	0.51
Ischemic core volume	0.99 (0.97–1.00)	**0.049**
Mismatch ratio	0.98 (0.90–1.07)	0.71
Favorable mTICI (≥2 b)	3.22 (1.25–8.26)	**0.015**
Space-occupying edema	0.60 (0.19–1.85)	0.37
Parenchymal hematoma	1.26 (0.54–2.95)	0.59
Other ICH	0.35 (0.09–1.38)	0.13

Multivariate binary logistic regression analyses were performed for the indicated parameters. Improvement was defined as 24-h decrease in NIHSS score > 4 compared to baseline; Bold *p* values indicate *p* < 0.05. Abbreviations: see Table 1; CI, confidence interval; OR, odds ratio.

## Data Availability

Anonymized study data are available from the corresponding author upon reasonable request.
